# Population Aging and Its Impact on Human Wellbeing in China

**DOI:** 10.3389/fpubh.2022.883566

**Published:** 2022-03-28

**Authors:** Meng-Yun Wang, Hsing-Chou Sung, Jie-Yi Liu

**Affiliations:** ^1^GXNU School of Politics and Public Administration, Guangxi Normal University, Guilin, China; ^2^The Department of Political Science, Tunghai University, Taichung, Taiwan; ^3^School of Architecture and Urban Planning, Suzhou University of Science and Technology, Soochow University, Suzhou, China

**Keywords:** ARDL, population aging, human wellbeing, health expenditure, China

## Abstract

Population aging is getting enlarged in the upcoming decades. Meanwhile, old-aged longevity and dependency are getting large due to improvement in life expectancy. In literature, it is claimed that old-aged dependency affects the wellbeing of society. Thus, the study intends to explore the impact of population aging on human wellbeing. The study adopts the Autoregressive Distributed Lag (ARDL) approach for empirical analysis by using time-series series data from 1990 to 2020. The study findings reveal that an increase in population aging reports a significant and decreasing impact on human wellbeing. However, an increase in health expenditure reports a significant and increasing impact on human wellbeing. Thus, China must pay attention to population aging to improve human health.

## Introduction

Population aging is an achievement of the advancement of society (1). Human societies have entered into a new stage with lower fertility and mortality rates due to social and economic development. When life expectancy increases, the governments have to make several social and economic adjustments to face the issue of population aging, particularly the effect of population aging on society's wellbeing and health services. The WHO adopted “Aging and Health” as the theme to celebrate the World Health Day of 2012 and elaborated the significance of good health for increasing life expectancy. Population aging gradually increases in middle and low-income economies (2). Due to effective public health and population policy, China is experiencing a very rapid increase in population aging. The population census of China revealed that the proportion of 65 and above-aged groups have increased from 5.57% in 1990 to 8.87% in 2010 (3).

According to the projections of the United States, the proportion of the 65 and above aged group will be 2 billion by the year 2050. Due to population aging in almost all economies of the world, there has been increasing concern about the impacts on human wellbeing and on the capability of economies to assist their old aged population. The old-aged people are relatively less economically active as compared to young people. Due to enlarging the proportion of old aged people, economic growth slows down, ultimately influencing the societies' overall wellbeing. These concerns are significantly intensifying in China, where almost 35 percent of the population will be expected to age 65 and above by 2050 (4). Population aging in China and other economic regions is rising due to several factors, such as increased life expectancy, low rate of fertility, and cumulative impact of past variations in death and birth rates (5). In China, the one-child policy has been the major reason behind the changing age structure of the population. Prolonged age is the key determinant in population aging. As long as population aging is rising in China, there is a need to identify such measures that can counteract the negative effects of population aging on the overall wellbeing of the whole society.

Several economies have designed policies that might alleviate economic issues related to population aging (6). These policies pursue raising the saving level age of retirement, increasing women's participation in the labor force, increasing incentives for higher education, liberalizing migration, and increasing child-care facilities. China has so far adopted the policy of increasing the legal retirement age. However, it is implausible to increase the saving rate in China as it is already very high. In the case of China, it is observed that increasing consumption policy will be relatively more effective in the coming years rather than increasing savings policy. However, China has attempted to redirect private, societal, and household sector savings to fund safe instruments for forthcoming retirements. Education has received widespread attention in China, and educational attainment has increased significantly, but a large population still lacks secondary education and technical training. Thus, improving the skills and education level of the Chines population could increase the wellbeing of people and can compensate for the dependency on working-age people (7).

Although there is no consensus regarding the magnitude and sign, the literature reported that there exists a most likely negative relationship between population aging and human development (8–10). The existing literature denoted various channels through which population aging influences human development. These include household savings patterns, household consumption patterns, human capital, and government sector social expenditure (11, 12). Most of the studies focused on the linkage between human development and population aging for developed economies. China's largest developing economy has the largest proportion of old-age people globally. Hence, China faces several social, economic, and human development issues.

The nexus between population aging and human development is still inconsistent in light of current literature. Some researchers argue that population aging negatively influences human development and wellbeing. For example, Modigliani and Cao (13) reported a negative association between population aging and human development. In contrast, some researchers denote that population aging promotes human capital accumulation and savings that are beneficial for human development in China (14, 15). Rapidly increasing population aging in China generates several health issues. For example, it is reported by the National Health and Fitness Commission of China that in 2018 among the 249 million people of China, almost 180 million people were facing non-communicable syndrome, around 40 million people were disabled, and the life expectancy was almost 68.71. China is facing high health pressure and social issues due to population aging, thus influencing the welfare of elderly people (6).

The association between population aging and health care services has been debated and explored quite extensively in existing literature (6, 16), but to the best of the authors' knowledge, the association between population aging and human wellbeing has not been explored yet. Based on these facts, the objective of this study is to assess the nexus between population aging and human wellbeing for China for the time period 1980 to 2020. This research will enrich the literature with an integrative and complex assessment of the association between population aging dilemma and human wellbeing by employing the Auto-regressive distributed lag (ARDL) approach. The study will help development practitioners in the implantation and formulation of such policies that help in improving human development, thus boosting human wellbeing. The study will also help economists prioritize investment for the human development sector.

## Model and Methods

Human development is mainly dependent on health, education, and level of income. Based on the importance of these three indicators United Nations (UN) has developed an index to represent human development based on three indicators such as Life Expectancy Index (LEI), Education Index (EI), and Income Index (II) (World Health Organization, 2015). Because of its comprehensive nature, this index is used widely to represent human wellbeing (17). The main objective of this analysis is to estimate the impact of population aging on the human development index in China. The literature has suggested the following model.


(1) $$\begin{array}{l} HDI_{t\;}=\alpha_{0\;}+\;\alpha_{1}PA_{t}+\alpha_{2}HE_{t}+\alpha_{3}ICT_{t}+\\ \;\alpha_{4}FD_{t}+\alpha_{4}Education_{t}+\mu_{t\;} \end{array}$$

Where human development index (HDI) is determined by population aging (PA), health expenditure (HE), information and communication technology (ICT), financial development (FD), average years of schooling (Education), and random error term (μ_*t*_). Most of the previous studies only focus on the long-run estimates; however, we aim to analyze both the short and long-run estimates in this analysis. However, the above model only provides the long-run estimates but we are interested both in the short and long-run estimates. Therefore, we will express the above model in an error correction model as specified below.


(2) $$\begin{array}{l} HDI_{t}=\;\gamma +\sum_{p=1}^{n1}\gamma_{1p\;}HDI_{t-p}+\\ \sum_{P=0}^{n2}\gamma_{2p\;}PA_{t-p}+\sum_{p=0}^{n3}\gamma_{3\;}HE_{t-p}+\;\sum_{p=0}^{n4}\gamma_{4p\;}ICT_{t-p}+\sum_{p=0}^{n5}\gamma_{5p\;}FD_{t-p}+\\ \sum_{p=0}^{n6}\gamma_{6p\;}Education_{t-p}+\pi_{1}HDI_{t-1}+\;\pi_{2}PA_{t-1}+\pi_{3}HE_{t-1}+\\ \pi_{4}ICT_{t-1}+\pi_{5}FD_{t-1}+\pi_{6}Education_{t-1}+\;\mu_{t} \end{array}$$

Specification (2) now resembles the autoregressive distributive lag order (ARDL) model of (18). The ARDL is considered superior as compared to other time series models in many aspects. For instance, in other time-series techniques, it is mandatory to check the stationarity of the variables, and the variables must be integrated in the same order (19). However, in the ARDL model, pre-unit root testing is not mandatory because it can account for integrating properties of the variables and can also analyse the variables whether they are stationary at level or first difference. Another benefit of this method is that it can simultaneously provide both short and long. In the above equation (2), the short-run estimates are those that are attached to the first difference variables, and the long-run estimates are connected to coefficients π_2_−π_6_ normalized on π_1_. However, these long-run estimates are considered valid only if they are cointegrated. To that end (18), proposed an F-test for the lagged level variables' joint significance and developed critical values for this test. If the calculated value of the F-test is greater than the critical values, this is confirmation of valid long-run results. Another important benefit of this method is that it performs well even if the sample size is small (20). Lastly, this method also includes a short-run dynamic process, which helps analyze any feedback effect, which is crucial for removing multicollinearity and endogeneity (21).

## Data

The study investigates the impact of population aging on human wellbeing in China. The study adopts time series data for the period 1990–2020. The detailed information regarding definitions and symbols of variables and descriptive statistics are provided in Table 1. Human wellbeing is captured through the human development index. Population above the age of 65 are considered for population aging. Health expenditures are measured in terms of the percentage of GDP. ICT and FD are measured through the ICT index and financial development index. Average years of schooling are used to measure education. The data for the human development index is collected from UNDP, while the data for the financial development index is taken from IMF, and the data for education is collected from Barro-Lee. However, the data for population aging, health expenditure, and ICT index is taken from the World Bank.

**Table 1:** Variables and data description.

**Variables**	**Definitions**	**Mean**	**Median**	**Maximum**	**Minimum**	**Std. Dev**.	**Skewness**	**Kurtosis**	**Sources**
HDI	HDI index	0.653	0.654	0.795	0.510	0.087	−0.023	1.717	UNDP
PA	Population ages 65 and above, total	18.41	18.40	18.94	17.99	0.264	0.296	2.237	World bank
HE	Current health expenditure (% of GDP)	4.283	4.273	5.350	3.491	0.540	0.315	1.974	World bank
ICT	ICT index	26.32	19.56	73.56	0.152	25.09	0.457	1.712	Authors' calculation
FD	Financial development index	0.466	0.446	0.654	0.275	0.117	0.130	1.746	IMF
Education	Average years of schooling	11.30	11.25	15.21	7.394	2.389	−0.011	1.681	Barro-Lee

## Results and Discussion

We aim to investigate the impact of population aging on the wellbeing of the people in China. Firstly, we have checked the stationary of the variables. Although the ARDL model allows us to include variables that are I(0), I(1), or a mixture of both, but I(2) variables are not allowed. Therefore, we have applied three stationary tests, including Augmented Dickey-Fuller (ADF), Dickey-Fuller Generalized Least Square (DF-GLS), and Phillips Perron (PP). The findings of these three tests are presented in Table 2. From Table 2, we can see that apart from Education, all variables are stationary at the first difference, i.e., integrated at first order. The results from the unit root test confirm that we can apply the ARDL model.

**Table 2:** Unit root test.

	**ADF**			**DF-GLS**			**PP**		
	**I(0)**	**I(1)**	**Decision**	**I(0)**	**I(1)**	**Decision**	**I(0)**	**I(1)**	**Decision**
HDI	−0.091	−4.785^***^	I(1)	0.235	−4.814^***^	I(1)	0.090	−4.775^***^	I(1)
PA	0.902	−2.635^*^	I(1)	0.042	1.725^*^	I(1)	1.023	−2.652^*^	I(1)
HE	−0.954	−5.201^***^	I(1)	−0.465	−5.320^***^	I(1)	−0.932	−5.329^***^	I(1)
ICT	0.754	−2.725^*^	I(1)	−1.902^*^		I(1)	1.365	−2.635^*^	I(1)
FD	−0.325	−5.302^***^	I(1)	0.452	−5.385^***^	I(1)	−0.521	−5.452^***^	I(1)
EDUCATION	−2.754^*^		I(0)	−1.689^*^		I(0)	−2.801^*^		I(0)

* *p < 0.1 and*

*** *p < 0.01*.

Table 3 presents the short and long-run estimates of the four models. First of all, we discuss the short-run estimates briefly. The estimates of ΔPA are significant and negative in three out of four models, confirming that population aging causes wellbeing to fall. Conversely, the estimates of ΔHE are significant and positive in three out of four models, implying that increased health expenditures improve the wellbeing of the people in China. However, the short-run estimates of ΔICT, ΔFD, and ΔEducation are mixed and inconclusive that is positive at some lags negative at other lags. Now the question arises, whether these short-run estimates persist in the long run or not?

**Table 3:** Long and short-run estimates of human wellbeing.

	**Model (1)**		**Model (2)**		**Model (3)**		**Model (4)**	
**Variable**	**Coefficient**	***t*-Stat**	**Coefficient**	***t*-Stat**	**Coefficient**	***t*-Stat**	**Coefficient**	***t*-Stat**
**Short-run**								
PA	−0.076	0.924	−0.059^***^	3.328	−0.057^***^	3.217	−0.040^***^	3.112
PA(−1)	0.128	1.432					1.098^***^	2.935
HE	0.005	1.503	0.008^**^	2.501	0.006^*^	1.727	0.006^*^	1.704
ICT			0.002^**^	1.960	0.002^**^	2.154	0.001	0.924
ICT(−1)			−0.002^**^	2.156	−0.002^**^	2.254	−0.001	0.813
ICT(−2)							0.002^*^	1.739
FD					0.044	0.968	0.016	0.441
FD(−1)							−0.017	0.517
FD(−2)							−0.047^*^	1.692
EDUCATION							0.020^***^	5.704
EDUCATION(−1)							−0.013^***^	2.872
**Long-run**
PA	−0.431^***^	7.855	−0.377^***^	3.160	−0.276^***^	2.617	−0.180^***^	2.668
HE	0.039	1.350	0.035^**^	2.189	0.032^*^	1.891	0.028^***^	2.597
ICT			0.004	0.043	0.005	0.441	0.006	0.607
FD					0.214^**^	1.986	0.088^**^	2.455
EDUCATION							0.009	0.484
C	−7.009^***^	7.886	−6.020^***^	2.893	−4.359^**^	2.429	−2.788	1.539
**Diagnostics**
F-test	12.35^***^		7.365^***^		6.258^***^		4.145^*^	
ECM(−1)	−0.376^*^	1.781	−0.356^***^	6.596	−0.347^***^	6.835	−0.309^***^	6.141
LM	1.365		1.752		1.254		0.608	
RESET	0.905		0.502		0.365		1.875	
CUSUM	S		S		S		S	
CUSUM-sq	S		S		S		S	

* *p < 0.1*;

** *p < 0.05; and*

*** *p < 0.01*.

To answer the above question, we turn our attention to Table 3. The estimates of PA are significantly negative in all four models, confirming that as people become old, it reduces the wellbeing of people in China. More precisely, a 1% increase in the population aging causes the wellbeing to fall by 0.431, 0.377, 0.276, and 0.180%, in the first, second, third, and fourth model, respectively. Our finding is also backed by Mohapatra et al. (22), who noted that population aging reduces health outcomes by increasing health expenditure in India. A similar finding is also found by Liu et al. (23) for Taiwan.

In general, our findings imply that with increasing the number of older adults in society, the people's overall wellbeing deteriorated. The wellbeing of society is explained by a combination of three indicators: education, life expectancy, and level of affluence. As more people cross 65 years of age or as the percentage of old people increases in society, the demand for health and social care increases (24). Besides, with the increasing proportion of older people government has to spend more on health care facilities and the incidence of health spending on the national exchequer also increases. Such phenomenon of the increasing number of older adults in the society will direct the flow of funds from education and other development activities to health-related activities that would reduce the level of education and income in the society. As a result, society's overall wellbeing is negatively affected because the wellbeing of society is measured by human development, which also includes indicators other than health.

On the other side, the estimates of HE are positively significant in three out of four models, which state that rising health expenditure improves the wellbeing of people in China. In numerical terms, we can say that as the health expenditures rise in China rise by 1%, they improve the wellbeing of people by 0.035% in the second model, 0.032% in the third model, and 0.028% in the fourth model. This is in line with the previous findings, which confirm the positive impact of health spending on economic growth, which is an indicator of the wellbeing of the society (25). Although, health care facilities are a basic necessity of the people in any society because there is a positive association between health and economic growth. Superior human health has a direct and positive impact on human capital development, which is connected to human welfare and, ultimately, economic growth (15). Hence, to attain sustainable and long-term economic development, spending on health care proves to be vital because a healthy person can perform his task more efficiently and contribute to society's wellbeing. However, the balance must be maintained between spending on health and spending on other factors (e.g., education) that also contribute to the development of human capital and, ultimately, the welfare of the whole population.

Similarly, the estimates attached to FD are significant and positive in both models, implying that a 1% increase in the financial development index improves wellbeing by 0.214 and 0.088%. However, the estimates of ICT and Education are positive but insignificant, confirming that both ICT and education do not significantly impact the wellbeing of the people of China.

To prove the legitimacy of our long-run results, we need to check the estimates of the F-test and ECM_t−1_ provided in Table 3 under diagnostics. The estimates attached to F-test are significant, confirming the valid long-run relationship between HDI, PA, HE, ICT, FD, and EDUCATION. Similarly, the estimates attached to ECM_t−1_ are significant and negative, and the estimated size represents the speed of convergence toward equilibrium. Apart from F-test and ECM_t−1_ tests, we have also presented a few other diagnostics, which strengthens our overall results. The first of these diagnostics is the Langrage Multiplier (LM) test which confirms that our models are free from the problems of serial correlation. Similarly, the Ramsey RESET test provides evidence that our models don't suffer from the issue of misspecification. Last but not least, CUSUM and CUSUM-sq provide information about the parametric stability of the model where “S” and “US” represent the stability and instability of the model, respectively. From seeing Table 3, we can endorse that our models are stable parametrically.

## Conclusion and Implications

The human development index is an important and well-known indicator of human wellbeing. This index comprises three different indicators, including education, health, and income, considered necessities of life. Various factors can affect human wellbeing, and in the literature, various studies are available that have tried to explore the determinants of human wellbeing. However, none of the studies have analyzed the impact of population aging on human wellbeing in China. Therefore, in this analysis, we have examined the population aging on human wellbeing in China.

The data used in the analysis is based on time series settings; therefore, we have checked the stationary of the variables. To that end, we have applied ADF, DF-GLS, and PP, confirming that our variables are a mixture of I(0) and I (1). Therefore, we have applied the ARDL model that can handle the variables with different orders of integration. Results of the ARDL model states that population aging has a negative and significant impact on human wellbeing in the long run in all four models. Similarly, the short-run estimates of population aging are significant and negative in three out of four models. Based on these estimates, we can state that population aging help to decrease human wellbeing both in the short and long run. The estimates of health expenditures are significant and positive in three out of four models both in the short and long run, implying that an increase in health expenditures causes human wellbeing to rise. Similarly, the financial development index estimates are significant and positive in both models, but only in the long run. Conversely, the estimates of ICT and Education have turned out to be insignificant. The F-test and ECM_t−1_ test have confirmed the cointegration among the long-run variables.

Our results imply that as people become old, they negatively impact the human development index, which is a sign of reducing human wellbeing. Therefore, policymakers should initiate special health and training programs for elderly people that would keep them healthy, active, skilled, and knowledgeable. As a result, elderly people can become an asset to society and contribute to society's overall wellbeing. Moreover, the policymakers should focus on increasing the health expenditures that would help to develop state-of-the-art health facilities, which are crucial for increasing human wellbeing in society. Furthermore, increasing health expenditures would also benefit the aging population, and they would become an active member of society, once again, and add value to society. Lastly, financial development can also prove vital for improving the wellbeing of the people because an active and vibrant financial sector can provide necessary funds for building health and education facilities, alongside spurring economic activity in the society. Therefore, policymakers should focus on developing the financial sector development to raise the level of human development.

The study consists of certain limitations. The major limitation is that the sample size is small and the study is limited to a single country that is China. In the future, research should be done in the case of panel economies. In future research, some other important dimensions of health and wellbeing should be added in models such as happiness, mental health, physical health, and social living standards. In the future, this study could be extended by including variables related to fiscal expenditures on social determinants such as education, health, old-age benefits, etc. The current study is limited to the national level of China, it is suggested that future research should be extended for provincial levels of China as well.

## Data Availability Statement

Publicly available datasets were analyzed in this study. This data can be found here: https://data.worldbank.org/.

## Author Contributions

M-YW: conceptualization, software, data curation, and writing- original draft preparation. H-CS: methodology, writing-reviewing, and editing. J-YL: visualization and investigation. All authors contributed to the article and approved the submitted version.

## Conflict of Interest

The authors declare that the research was conducted in the absence of any commercial or financial relationships that could be construed as a potential conflict of interest.

## Publisher's Note

All claims expressed in this article are solely those of the authors and do not necessarily represent those of their affiliated organizations, or those of the publisher, the editors and the reviewers. Any product that may be evaluated in this article, or claim that may be made by its manufacturer, is not guaranteed or endorsed by the publisher.
